# Genetic variations in three insecticide targets in the disease vector *Culex quinquefasciatus* from Mianyang, China: simultaneous detection of novel mutations RDL A296G and VGSC A1007T

**DOI:** 10.3389/fcimb.2026.1798952

**Published:** 2026-03-27

**Authors:** Xianying Wen, Hongwei Xie, Wenbo Deng, Mengmeng Dong, Meilin Tang, Zhengzheng Huang, Wanming Zhou, Xinghui Qiu

**Affiliations:** 1 Institute of Public Health Surveillance, Mianyang Center for Disease Control and Prevention, Mianyang, Sichuan, China; 2 State Key Laboratory of Animal Biodiversity Conservation and Integrated Pest Management, Institute of Zoology, Chinese Academy of Sciences, Beijing, China

**Keywords:** acetylcholinesterase (AchE), AChE G119S mutation, Culex quinquefasciatus, GABA receptor RDL subunit, RDL A296G mutation, VGSC A1007T mutation, voltage-gated sodium channel (VGSC)

## Abstract

*Culex quinquefasciatus* is a major vector of filariasis and Japanese encephalitis in China. The application of insecticides is a regular measure for prevention and control of such mosquito-borne diseases. However, insecticide resistance, often caused by target-site mutations, threatens control efforts. In Mianyang City, a region previously reporting high pyrethroid resistance, the current genetic basis of resistance remains unclear. To address this gap, resistance-related mutations in three key insecticide targets, including acetylcholinesterase (AChE, encoded by *ace1*, conferring organophosphate/carbamate resistance), GABA receptor RDL subunit (encoded by *rdl*, conferring dieldrin/fipronil resistance), and voltage-gated sodium channel (VGSC, *encoded by vgsc*, conferring pyrethroid/DDT resistance), in seven field populations of *Cx. quinquefasciatus* collected across Mianyang were simultaneously screened by gene sequencing. The results showed that the known AChE G119S mutation was present at low frequencies (1.25% to 5.1%). Two RDL mutations, the previously reported A296S and a novel A296G, were widely detected at appreciable frequencies. In the *vgsc* gene, the knockdown resistance allele L1014F was dominant (overall frequency 88%), and a novel A1007T mutation emerged on the L1014F allele. This study provides the first comprehensive snapshot of co-existing target-site resistance mutations in *Cx. quinquefasciatus*. The simultaneous presence of mutations across three distinct target mechanisms, including two first-reported mutations (RDL-A296G and VGSC-A1007T), signals a multifaceted and evolving resistance landscape. These findings highlight an urgent need for resistance monitoring and management in this important disease vector.

## 1 Introduction

*Culex quinquefasciatus* Say (Diptera: Culicidae), commonly known as the southern house mosquito, is widely distributed in tropical and subtropical regions of the world. This mosquito species is an important vector of lymphatic filariasis, West Nile fever, Japanese encephalitis and Saint Louis encephalitis. Historically, the control of mosquito vectors including *Cx. quinquefasciatus* has generally relied on insecticides, including carbamates, organochlorines, organophosphates, and pyrethroids. However, the extensive use of these traditional chemical insecticides has led to the development of resistance in numerous mosquito species worldwide (Liu et al., 2024; Mathias et al., 2024; Huang et al., 2025). *Cx. quinquefasciatus* resistance against insecticides was reported from different regions of the world and is increasing (Abbasi and Daliri, 2024).

Understanding the mechanisms underlying insecticide resistance is crucial for resistance monitoring and management. Among the documented insecticide resistance mechanisms, target insensitivity caused by mutations in target proteins of insecticides is a common and important one (Abbasi and Daliri, 2024; Hemingway et al., 2004; Liu, 2015). For *Cx. quinquefasciatus*, the resistance-conferring G119S mutation in the acetylcholinesterase1 (AChE, encoded by *ace1* gene, conferring resistance to organophosphates and carbamates) (Weill et al., 2003; Low et al., 2013; Liu et al., 2023), A296S in the γ-aminobutyric acid (GABA) receptor RDL (resistant to dieldrin) subunit (encoded by *rdl* gene, conferring resistance to dieldrin and fipronil) (Tantely et al., 2010; Amelia-Yap et al., 2020), and L1014F/S in the voltage-gated sodium channel (VGSC, conferring knockdown resistance to pyrethroids and DDT) (Xu et al., 2005; Rai and Saha, 2022; Liu et al., 2023; Huang et al., 2025; Chamnanya et al., 2025) have been documented. Recently, two novel concomitant mutations L932F and I936V in VGSC were reported in a Brazilian strain of *Cx. quinquefasciatus*, associated with a high pyrethroid resistance ratio value and present in trans configuration to the classical L1014F (Sugiura et al., 2021).

**Figure 1:**
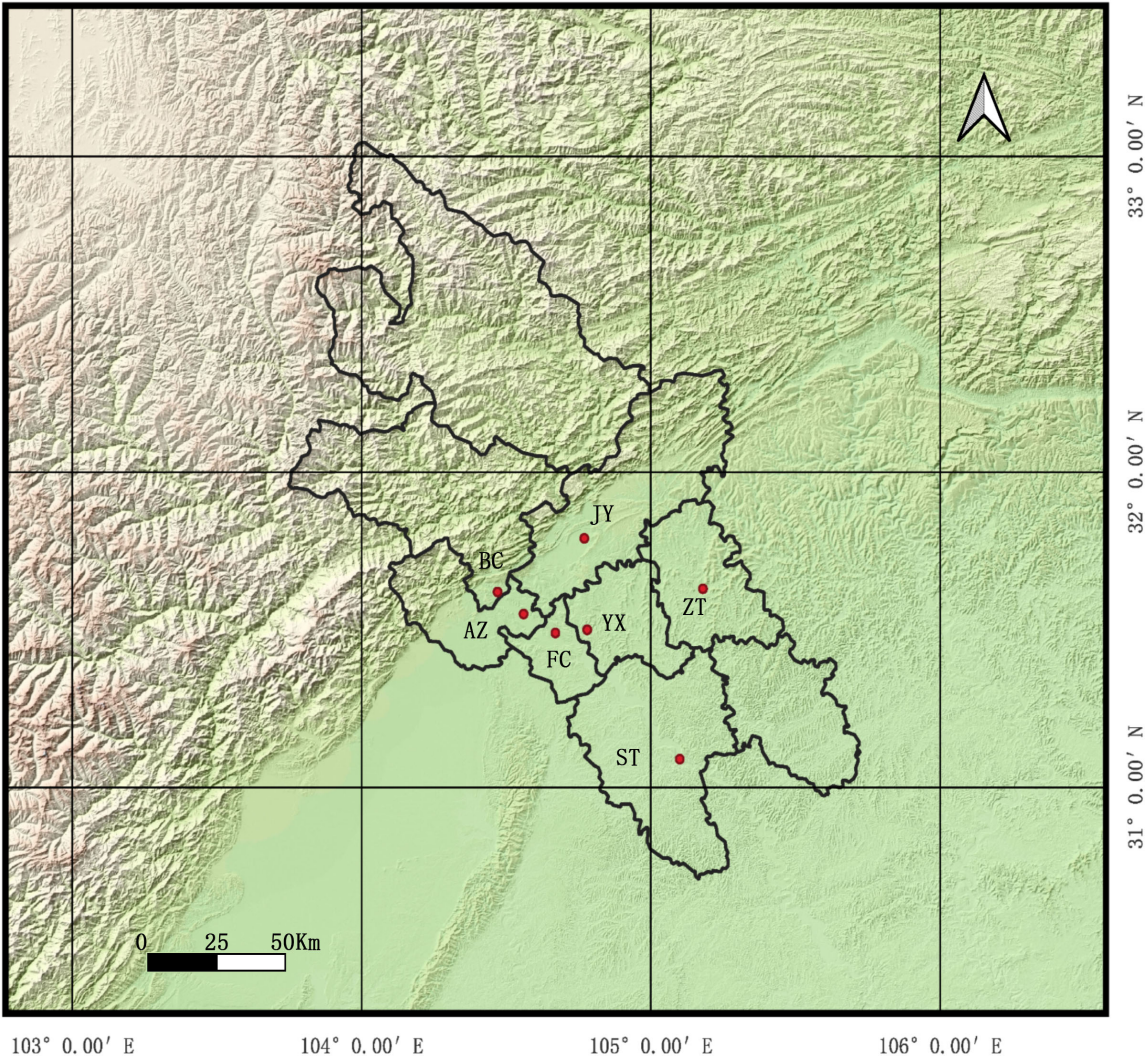
Sampling locations of *Culex quinquefasciatus* in Mianyang City. The base map was generated using QGIS 3.34.3.

Mianyang City is a major prefectural-level city nestled in northwestern Sichuan Basin of China with a population of approximately 5.25 million. Its natural environment is defined by the Fu River flowing through the urban center and the mountainous terrain of the Longmen Mountains to the west (**Figure 1**). The city is located within the north subtropical mountainous humid monsoon climate zone with distinct seasons, featuring fertile plains in the east. Its natural and social environment—characterized by mild winters, abundant rainfall, and substantial population mobility—is conducive to mosquito breeding and the spread of mosquito-borne diseases.

Given that *Cx. quinquefasciatus* is one of the most abundant mosquito species and the vector of filariasis and Japanese encephalitis in Mianyang, it is of great significance to understand its status of resistance to commonly used chemical insecticides. In a survey ten years ago, resistance to beta⁃cypermethrin (2925-fold), deltamethrin (90.59-fold), dichlorvos (9.85-fold), and fenobucarb (3.9-fold) was reported in larvae collected from Beichuan county of Mianyang (a disaster zone of the 5·12 Wenchuan Earthquake in 2008 where pyrethroid and organophosphorus insecticides were intensively used) in 2013 (Hu et al., 2015). However, little was known about the current status in other locations and the involved genetic mutations associated with insecticide resistance in *Cx. quinquefasciatus* in this region. To address this knowledge gap and to provide critical data for local vector control strategies, this study aimed to simultaneously investigate the presence and frequency of mutations in three major insecticide target genes (*ace1*, *rdl*, and *vgsc*) in field populations of *Cx. quinquefasciatus* from Mianyang. Our data reveal, for the first time in this species, the A296G mutation in RDL and the novel A1007T mutation in VGSC, alongside a detailed assessment of the established G119S in AChE and L1014F/S mutations in VGSC. These findings should facilitate the diagnosis and improve the understanding of resistance evolution in this medically important species.

## 2 Materials and methods

### 2.1 Mosquito samples

Samples of *Cx. quinquefasciatus* were collected using UV lamp-traps (LTS-M02, Kongfu Dude) from seven locations in Mianyang City from June to September 2024, including Fucheng (FC), Youxian (YX), Beichuan (BC), Anzhou (AZ), Jiangyou (JY), Zitong (ZT) and Santai (ST) (**Figure 1**). Species identification was initially conducted through morphological examination. The accuracy of species identification was confirmed molecularly based on the size of PCR amplicon of the *rDNA-ITS2* gene according to the method of Song et al. (2003). The samples that carry the novel mutations were further confirmed by amplicon sequencing of the *cytochrome c oxidase subunit I (COI)* gene generated by PCR using the universal primers LCO1490 (GGTCAACAAATCATAAAGATATTGG) and HCO2198 (TAAACTTCAGGGTGACCAAAAAATCA).

### 2.2 DNA extraction

The genomic DNA of individual mosquitoes was isolated according to the protocol described in Xie et al. (2025). Briefly, the sterile water-washed head and thorax of individual adults were placed in a 1.5 mL EP tube with 50 µL of lysis buffer (100mM TrisHcl, pH8.0, 10 mM EDTA, 50 mM NaCl and 1% SDS), ground using TGrinder OSE-Y30 (Tiangen, China), and supplemented with additional 300 µL of lysis buffer. Then the sample was mixed with five µL protease K (20 mg/mL), and incubated at 60 °C for one hour. After that, 40 µL of 8 M potassium acetate solution was added into the tube, and left on ice for 10 min. After the mixture was centrifuged at 14000g for 30 min, 320 µL of supernatant was taken to a new centrifuge tube. Then, 640 µL of chilled anhydrous ethanol were added into the supernatant, and the samples were kept at room temperature for 20 min after mixing, followed by centrifugation at 14000 g for 20 min. The pellet was resuspended using 600 µL of 70% ethanol, and centrifuged at 8000 g for 15 min. The DNA pellet was air-dried, dissolved in ~ 30 µL of sterile water, and stored at 4°C or -20°C.

### 2.3 PCR amplification of fragments of insecticide target-encoding genes

A fragment of each insecticide target-encoding gene, where the well-recognized resistance-related mutation resides, was generated *via* polymerase chain reaction (PCR). Primers used for *ace1, rdl* and *vgsc* gene were CqAce1-119 (F: 5’-GCGCGAGCATATCCATAGCACT-3’, R: 5’-TCTGATCAAACAGCCCCGCGT -3’; Yanola et al., 2015), Cq-rdl296 (F: 5’-CAGTTTGTACGTTCGATGGGT-3’, R: 5’-GGCAAATACCATGACGAAGCA-3’; this study), and Cq-V1014 (F: 5’-GGTGGAACTTCACCGACTTC-3’, R: 5’-GGACGCAATCTGGCTTGTTA -3’; Yanola et al., 2015), respectively. All reactions were carried out in a total volume of 20 µL, containing 10 µL of 2 × Es Taq MasterMix (CWBIO, Beijing, China), 0.5 µL of each primer, 8 µL of ddH2O, and 1 µL of DNA template (50–200 ng). The PCR programs were set as: initial denaturation at 94 °C for 2 min, followed by 35 cycles of 30s at 94 °C, 30s at 58 °C, and 30s at 72 °C, with final extension of 2 min at 72 °C.

### 2.4 DNA sequencing and sequence analysis

The PCR products were detected by electrophoresis on a 1% agarose gel. The positive amplicons were Sanger sequenced after purification by BGI Company (Beijing, China). DNA sequences obtained by Sanger sequencing were manually checked and both-end trimmed. Haplotypes were identified by directly reading from homozygotes or heterozygotes with one polymorphic site. All confirmed sequences were analyzed using Muscle 3.8 (Edgar, 2004), and MEGA 7 (Kumar et al., 2016). The organization of intron and exon was identified based on the whole genome shotgun sequence NC_051863.1 for *ace1* and *rdl*, and NC_051862.1 for *vgsc*. The GenBank accession numbers (will be publicly released upon publication of this paper) for the haplotypes described in this study were PX893157 to PX893165 for *vgsc*, PX893166 to PX893181 for *ace1*, and PX893182 to PX893186 for *rdl* (**Supplementary Table S1**).

## 3 Results

### 3.1 Genetic variations in the *ace1* gene

**Figure 2:**
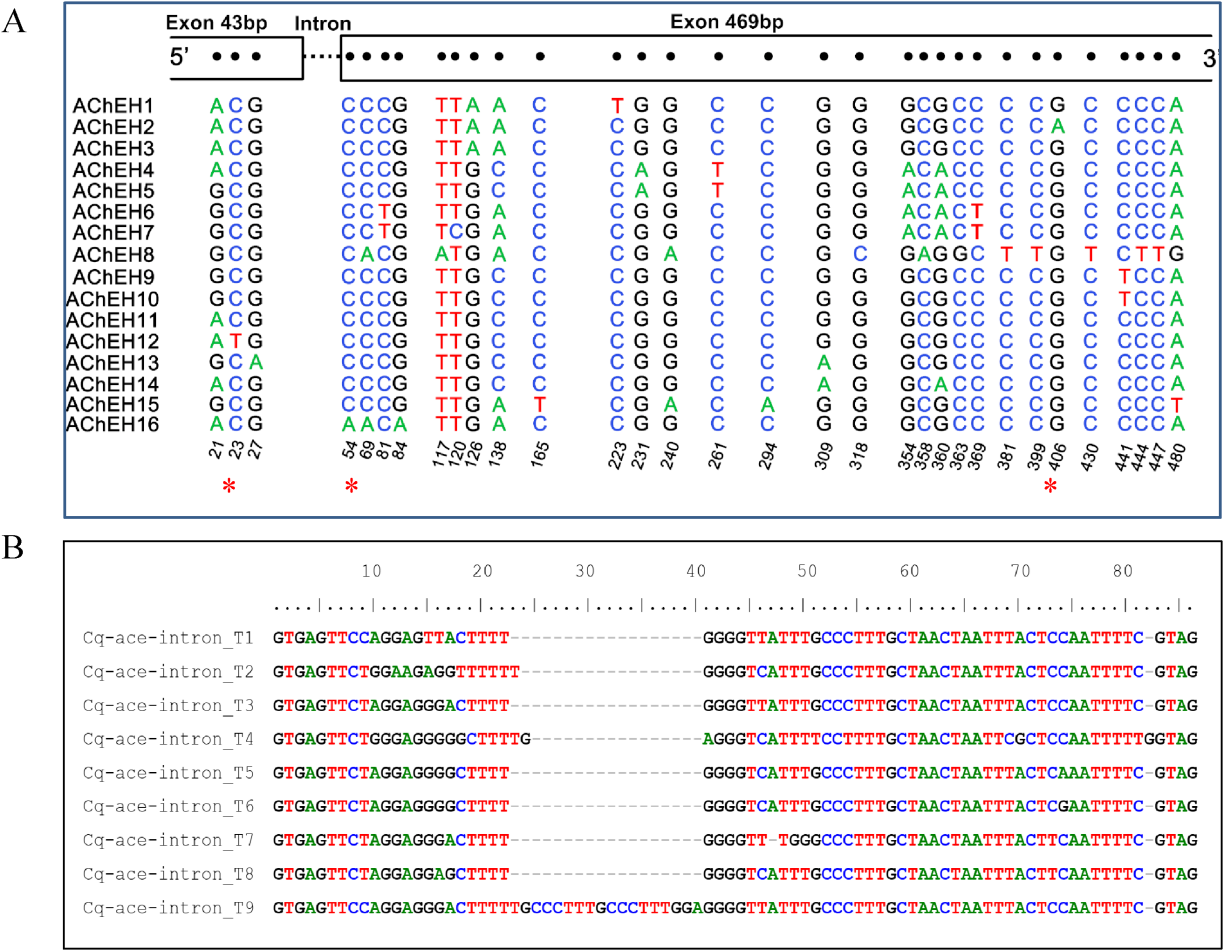
Nucleotide polymorphism of *ace1* haplotypes identified in this study. **(A)** shows the single nucleotide polymorphic sites identified in exons; * indicates nonsynonymous variation. **(B)** is an alignment of nine types of intron 3.

A fragment of the *ace1* gene covering partial exon 3, the complete intron 3 and partial exon 4 was amplified from a total of 308 samples. Within the exon region, 32 polymorphic sites were detected with three of them being nonsynonymous. These nonsynonymous variations resulted in three amino acid substitutions (T/I, D/E, G/S), including the well-known resistance-conferring G119S mutation (**Figure 2A**). Nine distinct intron types were identified, varying in length (66, 67, 68, 70, and 85-bp) and also exhibited sequence variation at the nucleotide level (**Figure 2B**).

At the resistance-related 119 locus (AChE-119), four different codons (GGA, GGC, GGT and AGC) were identified. Four individual genotypes and two alleles were detected (**Table 1**). The resistant allele (119S, encoded by AGC) was present in the heterozygous form, and distributed across all the investigated populations at a frequency ranging from 1.25% to 5.1% (**Table 1**).

**Table 1:** Genotype distribution and allele frequencies (%) at the *ace1–*119 locus in seven *Culex quinquefasciatus* populations in Mianyang City of Sichuan Province, China.

Population	N	Genotype				Allele	
		GGC	GGC/GGA	GGC/GGT	GGC/AGC	119G	119S
		(119GG)	(119GG)	(119GG)	(119GS)		
AZ	51	90.2	1.96	0	7.84	96.08	3.92
BC	40	97.5	0	0	2.5	98.75	1.25
FC	48	93.75	0	2.08	4.17	97.92	2.08
JY	47	95.74	0	0	4.26	97.87	2.13
ST	49	87.8	0	0	10.2	94.9	5.1
YX	28	96.43	0	0	3.57	98.21	1.79
ZT	45	93.33	2.22	0	4.44	97.78	2.22
Total	308	93.51	0.65	0.32	5.52	97.24	2.76

**Figure 3:**
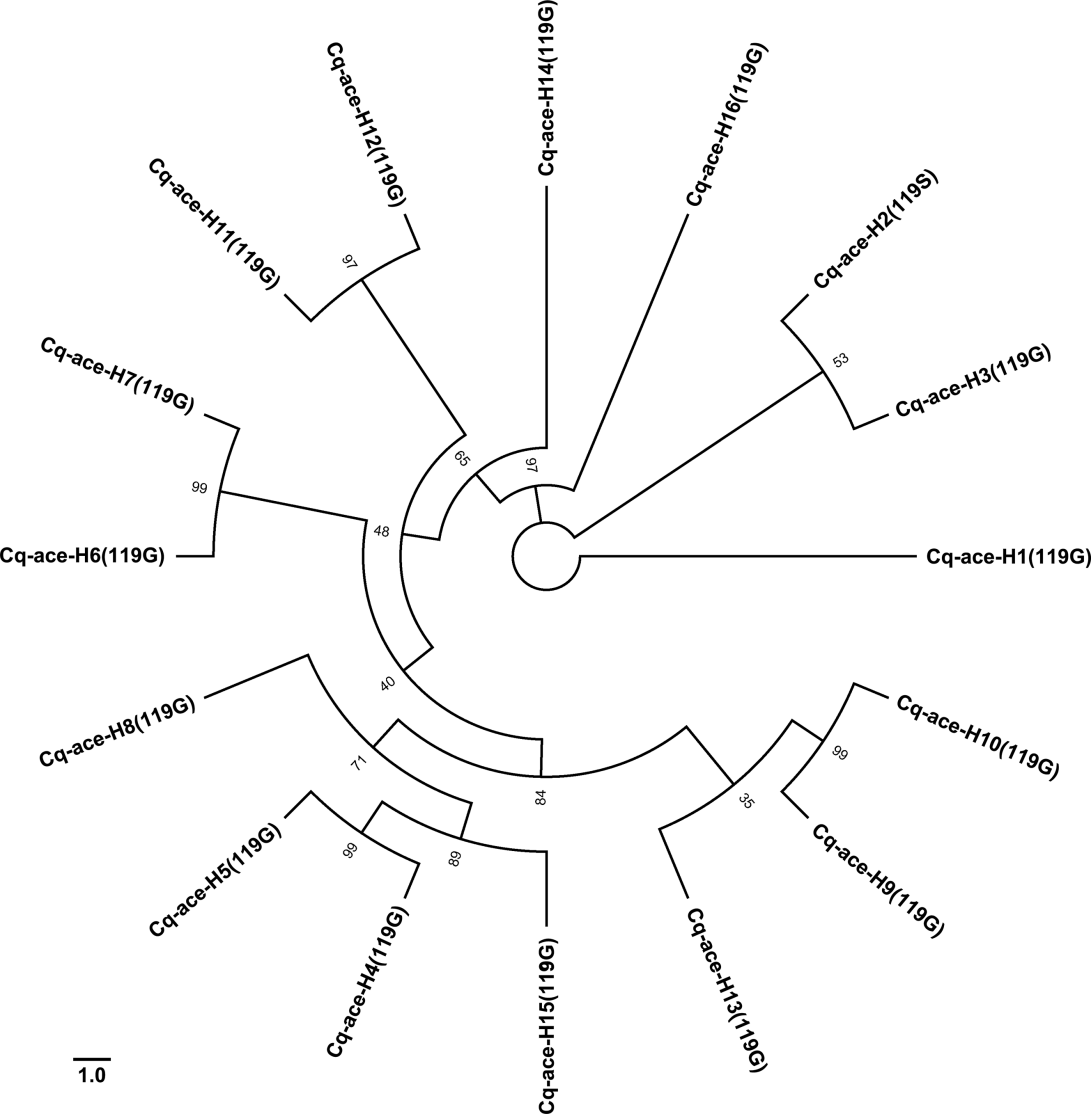
Maximum Likelihood tree of the *ace1* haplotypes identified in this study. Phylogenetic analysis was conducted in IQ-tree2 (Minh et al., 2020). The best-fitting evolutionary model was selected in IQ-tree2 with option “-m MFP”: TPM2+F+I for the exon and K3Pu+F for the intron. The tree with the highest log likelihood (-1168.891) is shown. Node supports were estimated using 10,000 ultrafast bootstrap replicates. This analysis involves 16 nucleotide sequences. There was a total of 579 positions in the final dataset.

Among the 16 identified haplotypes, only Cq-ace-H2 carried the resistance-associated G119S mutation (**Figure 2**). Phylogenetic analysis (**Figure 3**) revealed that Cq-ace-H2, -H3, and -H1 were clustered within the same clade and shared identical intron sequences, suggesting that the resistant haplotype (H2) might originate from either H1 or H3. Furthermore, haplotypes with glycine at position 119 were distributed across distinct clades, highlighting the substantial genetic diversity of the *ace1* gene.

### 3.2 Genetic variations in the *rdl* gene

**Figure 4:**
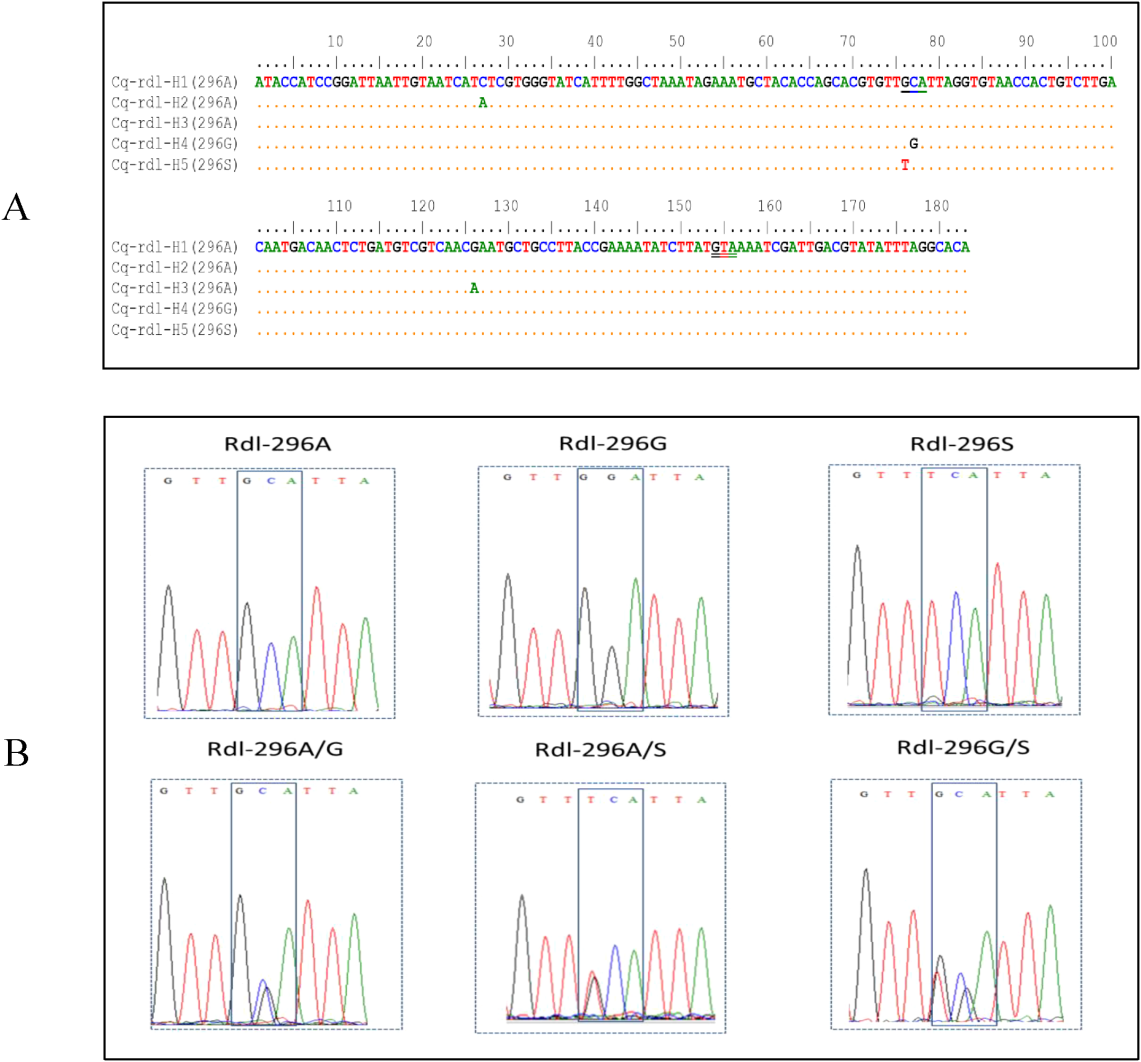
Nucleotide sequence alignment of the *rdl* haplotypes identified in this study **(A)**, and the representative DNA sequencing chromatogram of the amplicons of the *rdl* gene showing the genotype at the 296 locus **(B)**.

Analysis of a 183-bp gene fragment (comprising exclusively exon 7 sequence) identified five *rdl* haplotypes (**Figure 4A**). Four nucleotide polymorphic sites were detected: a synonymous C/A substitution at position 27, non-synonymous G/T and C/G substitutions at positions 76 and 77, respectively, and a synonymous G/A substitution at position 126. These correspond to two amino acid replacements at residue 296: A296G (GCA to GGA) and A296S (GCA to TCA).

At the 296 locus of the *rdl* gene, six genotypes and three alleles were identified in the samples (**Figure 4B**, **Table 2**). The wild-type homozygote was predominant, with a frequency ranging from 57.69% (YX) to 90.57% (FC) (**Table 2**). All the three alleles were distributed across all seven populations. Notably, the frequency of the wild-type allele (296A) was consistently above 85% overall, followed by the resistant 296G, while another resistant allele (296S) was the least frequent.

**Table 2:** Genotype distribution and allele frequencies (%) at the *rdl* 296 locus in seven *Culex quinquefasciatus* populations in Mianyang City of Sichuan Province, China.

Population	N	Genotype						Allele			
		GCA	GGA	TCA	GCA/GGA	GCA/TCA	GGA/TCA	296A	296G	296S	296G+296S
		(296AA)	(296GG)	(296SS)	(296AG)	(296AS)	(296GS)				
AZ	51	66.7	3.92	0	19.61	9.8	0	81.37	13.73	4.9	18.63
BC	50	72	0	0	16	10	2	85	9	6	15
FC	53	90.57	0	0	7.54	1.89	0	95.28	3.77	0.94	4.71
JY	46	76.09	0	4.35	15.22	4.35	0	85.87	7.61	6.52	14.13
ST	48	68.75	2.08	0	22.91	6.25	0	83.33	13.54	3.13	16.67
YX	26	57.69	0	0	23.08	19.23	0	78.85	11.54	9.62	21.15
ZT	49	79.59	2.04	0	14.29	2.04	2.04	87.76	10.2	2.04	12.24
Total	323	74.3	1.23	0.62	16.41	6.81	0.62	85.91	9.75	4.33	14.09

### 3.3 Genetic variations in the *vgsc* gene

**Figure 5:**
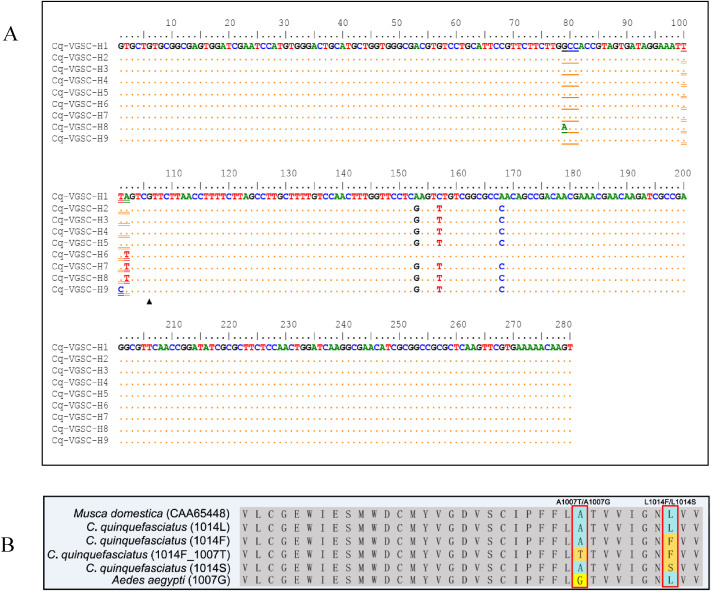
Nucleotide sequence alignment of the *vgsc* haplotypes identified in this study, and the codon 1007 is marked by single underline, and codon 1014 by double underline **(A)**. Multiple alignments of the partial deduced amino acid sequences of the VGSC multiple alignments of the partial deduced amino acid sequences of VGSC from *Culex quinquefasciatus*, *Musca domestica* and *Aedes aegypti* **(B)**.

The analyzed DNA sequences of the *vgsc* fragment consisted of partial exon 20 (107 bp), a complete intron (intron 20, ~ 327 bp) and partial exon 21 (175 bp). Six nucleotide polymorphic sites were detected in the exon 20, leading to three amino acid substitutions: A1007T, GCC to ACC), L1014F (TTA to TTT), and L1014S (TTA to TCA) (**Figure 5**). Considerable variations were observed in the intron, including base substitution, insertion and deletion (Data not shown).

**Figure 6:**
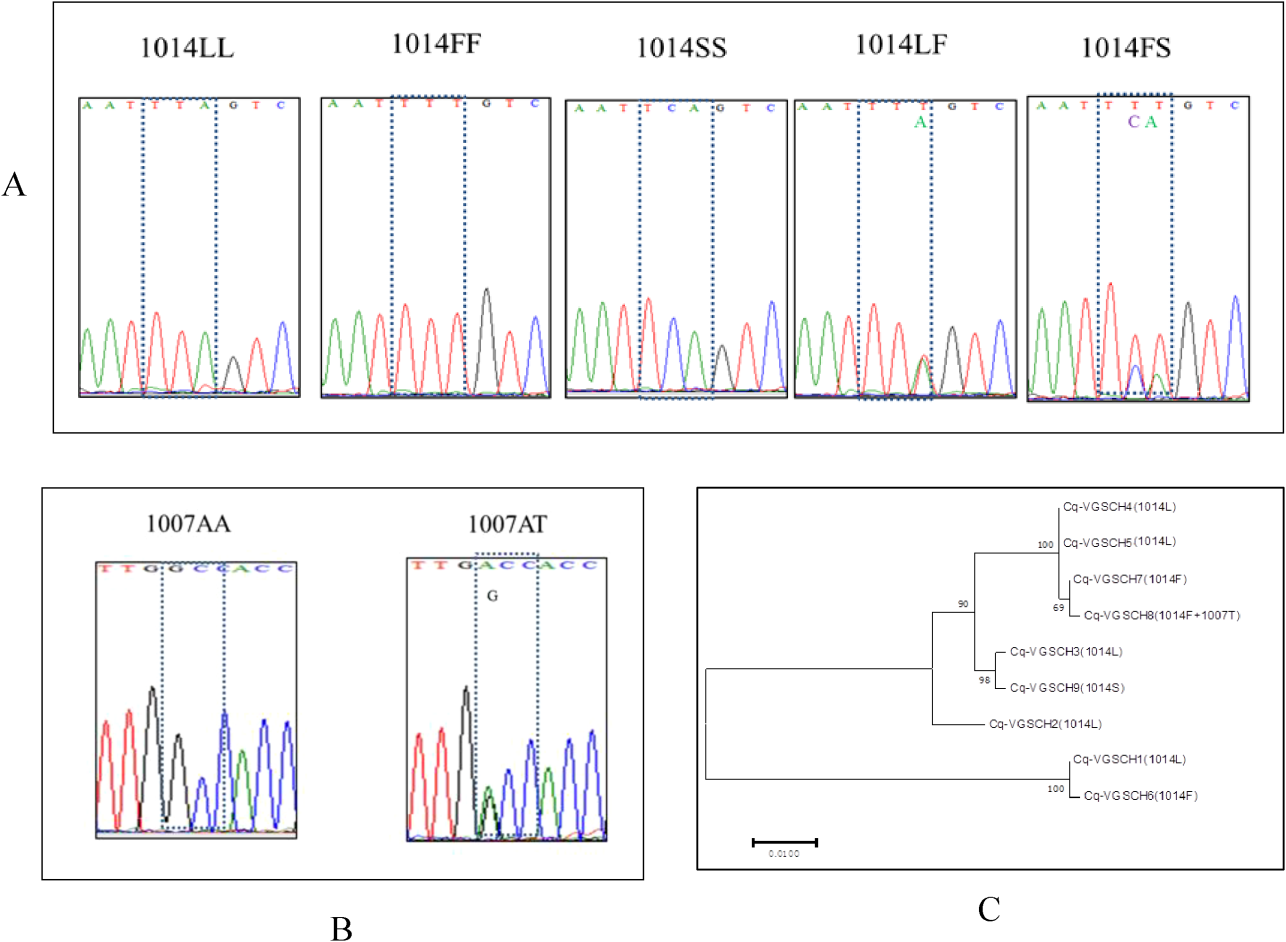
Chromatograms showing the genotypes at locus 1014 **(A)** and locus 1007 **(B)**, and the molecular phylogenetic tree **(C)** of the nine *vgsc* haplotypes detected in this study. The molecular phylogenetic tree was inferred by using the Maximum Likelihood method based on the Tamura-Nei model (Tamura and Nei, 1993). The tree was drawn to scale, with branch lengths measured in the number of substitutions per site. The analysis involved 9 nucleotide sequences. There was a total of 621 positions in the final dataset. Evolutionary analyses were conducted in MEGA7 (Kumar et al., 2016).

Focusing on the classical *kdr-*related locus (VGSC-1014), five genotypes were detected in our samples (**Figure 6A**). Resistant homozygote 1014FF was the predominant genotype, with a frequency ranging from 69.05% (BC, ZT) to 97.14% (JY) (**Table 3**). Among the three identified alleles, the resistant 1014F allele reached an overall frequency of 88%, whereas the 1014S allele was present in five of the seven populations at lower frequencies (**Table 3**).

**Table 3:** Genotype distribution and allele frequencies (%) at the *vgsc*1014 locus in seven *Culex quinquefasciatus* populations in Mianyang City of Sichuan Province, China.

Population	N	Genotype					Allele			
		TTA	TTT	TCA	TTA/TTT	TTT/TCA	1014L	1014F	1014S	1014F+1014S
		(1014LL)	(1014FF)	(1014SS)	(1014LF)	(1014FS)				
AZ	50	0	90	0	2	8	1	95	4	99
BC	42	4.76	69.05	4.76	11.9	9.52	10.71	79.76	9.52	89.28
FC	39	0	89.74	0	10.26	0	5.13	94.87	0	94.87
JY	35	0	97.14	0	2.86	0	1.43	98.57	0	98.57
ST	49	6.12	73.5	0	16.32	4.08	14.29	83.67	2.04	85.71
YX	30	0	73.33	3.33	23.33	0	11.67	85	3.33	88.33
ZT	42	7.14	69.05	2.38	19.05	2.38	16.67	79.76	3.57	83.33
Total	287	2.79	80.14	1.39	11.85	3.83	8.7	88	3.3	91.3

In addition to the mutation at position 1014, a novel amino acid replacement was identified at residue 1007 in two of the 287 examined individuals (one in JY, and another in ZT). This mutation co-existed with 1014F, and was present only in the heterozygous form (**Figure 6B**).

Nine *vgsc* haplotypes were identified in our samples, among which five were of the 1014L type (wild), three were of the 1014F type (resistant), and one was of the 1014S type (resistant). Molecular phylogenetic analysis (**Figure 6C**) shows that the 1014F types cluster into two distinct evolutionary branches, suggesting that the 1014F mutation in Mianyang populations of *Cx. quinquefasciatus* may have independent evolutionary origins.

## 4 Discussion

This survey of seven *Cx. quinquefasciatus* populations in Mianyang revealed a concerning landscape of target-site resistance mutations across three major insecticide targets. The most alarming finding is the near-fixation of knockdown resistance (*kdr)* mutations in the voltage-gated sodium channel (VGSC), with the resistance alleles (1014F + 1014S) exceeding 90% frequency, and homozygous 1014FF individuals exceeding 80% overall (**Table 3**). This suggests a high risk of pyrethroid failure in Miangyang. This concern is supported by Yanola et al. (2015), who observed 0% mortality following exposure to WHO 0.05% deltamethrin paper for this specific genotype. In contrast, the G119S mutation in acetylcholinesterase remained at low frequencies and existed in heterozygous form, indicating that organophosphate/carbamate resistance mediated by this target site mutation is currently less dominant. One possible explanation for the low frequency is the high fitness cost associated with the G119S substitution (Raymond et al., 2001). A similar pattern (near fixed VGSC L1014F and rare AChE G119S) was also observed in field populations of *Cx. quinquefasciatus* from southern Sichuan region of China (Liu et al., 2023). Notably, resistance alleles in the GABA receptor (RDL) reached non-negligible frequencies (> 10% in six of seven populations). The prevalent co-existence of VGSC and RDL mutations highlights a serious, multi-mechanistic threat to insecticide efficacy, necessitating immediate resistance management strategies.

In Mianyang, organochlorines and organophosphates were historically used in agriculture and public health. Currently, vector control strategies heavily rely on pyrethroids, while agriculture extensively utilizes pyrethroids, neonicotinoids and diamides. This pattern of insecticide usage in Mianyang aligns generally with our genetic findings. The near-fixation of *kdr* mutations (VGSC) in all populations strongly correlates with the intensive and prolonged use of pyrethroids in both public health and agriculture. Conversely, the low frequency of the AChE G119S mutation may reflect the relatively less and more targeted application of organophosphates in recent decades, or as Raymond et al. (2001) suggested, a high fitness cost preventing its fixation despite intermittent exposure. The non-negligible frequency of RDL mutations in most populations likely represents a legacy effect from the historical use of cyclodienes and fipronil, although other factors could not be excluded. The co-existence of VGSC and RDL mutations suggests that these *Cx. quinquefasciatus* populations have been shaped by a layered history of multiple insecticide application.

A key novel finding of this study is the first report of the A1007T mutation in the VGSC of *Cx. quinquefasciatus*, co-existing with L1014F. Intriguingly, a mutation at the same residue (A1007G) has been associated with pyrethroid resistance and also co-existed with a well recognized *kdr* F1534C mutation in *Ae. aegypti* from Southeast Asia (Vietnam and Malaysia) (Lien et al., 2018; Zuharah and Sufian, 2021; Akhir et al., 2022). A recent functional study showed that A1007G had a distinct effect on sodium channel sensitivity to Type I (permethrin and bifenthrin), and synergistic effects were observed between mutations A1007G and F1534C, but not to Type II pyrethroids (deltamethrin and cypermethrin) (Chen et al., 2025). Given that the residue 1007 is part of the pyrethroid receptor site 2 (Chen et al., 2025), we hypothesize that A1007T may confer a similar, possibly synergistic, effect on channel insensitivity in combination with L1014F. Although currently at a low frequency and narrowly distributed, the emergence of this dual-mutant haplotype (A1007T + L1014F) warrants urgent functional validation and surveillance, as it may represent a new evolutionary pathway to enhanced pyrethroid resistance.

In mosquitoes, the amino acid substitution (A to S) at position 296 (equivalent to residue 301 of RDL in the *Drosophila melanogaster*) in the second membrane-spanning domain has been associated with dieldrin resistance in several species including *Cx. quinquefasciatus* (Tantely et al., 2010). This conserved mutation A296S has been detected in other mosquito species such as *An. sinensis* (Qian et al., 2021) and *Cx tritaeniorhynchus* (Liu et al., 2023; Xie et al., 2025) in Sichuan Province of China. Another substitution at the same position (A296G) has been documented to be associated with dieldrin resistance in *An. gambiae* and *An. arabiensis* (Du et al., 2005), and in *An. funestus* (Wondji et al., 2011). Interestingly, both A296S and A296G mutations were detected in *Cx. quinquefasciatus* populations from Mianyang. To the best of our knowledge, A296G mutation was discovered for the first time in this species. Notably, A296G was found at a frequency higher than that of A296S (**Table 2**). The persistence and diversification of RDL mutations long after the banning of cyclodienes (e.g., dieldrin) strongly suggest subsequent selection pressure from other agriculturally used insecticides that act on the GABA receptor, such as fipronil and isoxazolines. While we could not directly confirm this specific selection pressure due to the lack of data on the application of such insecticides in the study area, this observation underscores the potential for cross-resistance to occur within these insecticide classes in *Cx. quinquefasciatus*.

Our study documents not only the co-existence of mutations across different target genes but also the co-presence of multiple resistance alleles at the same locus (i.e., L1014F and L1014S in VGSC; A296S and A296G in RDL) in field populations of *Cx. quinquefasciatus* in Mianyang. This genetic complexity suggests that local mosquito populations are undergoing dynamic and multifaceted adaptive evolution under varying insecticide selection pressure. The co-existence of multiple mutations at the same locus within the same populations presents an evolutionary phenomenon whose toxicological and fitness significance merits further studies. From an operational perspective, this complexity severely limits the utility of insecticide rotation based on mode of action, as multiple targets are already compromised.

This study provides a critical genotypic snapshot but has limitations. First, it focuses solely on target-site resistance; other mechanisms such as metabolic resistance and cuticular penetration reduction likely contribute to the overall phenotype. Second, corresponding phenotypic bioassay (e.g., WHO tube tests) data are absent, thus we could not definitively link the discovered genotypes (especially the novel A1007T and A296G) to resistance levels. Future work should: (1) functionally characterize the novel mutations *via* electrophysiology or toxicity bioassay; (2) integrate phenotypic bioassays with genotypic monitoring; (3) explore other potential mechanisms responsible for the field-evolved insecticide resistance beyond target site insensitivity, such as metabolic resistance mediated by cytochrome P450, in this medically important species.

## 5 Conclusion

This study reveals a comprehensive landscape of target-site insecticide resistance mutations in field populations of Cx. *quinquefasciatus* from Mianyang. We report, for the first time in this species, two novel resistance mutations: VGSC A1007T and RDL A296G. Our findings not only demonstrate the genetic complexity that compromises conventional insecticide rotation strategies and underscores an urgent need to establish an integrated resistance monitoring and management program. This work expands the known genetic repertoire of insecticide resistance in insects, and provides critical data for evidence-based control of this important mosquito vector.

## Data Availability

The datasets presented in this study can be found in online repositories. The names of the repository/repositories and accession number(s) can be found below: https://www.ncbi.nlm.nih.gov/genbank/, PX893157-PX893186.
