# Supplementary material for: Genetic variations in three insecticide targets in the disease vector Culex quinquefasciatus from Mianyang, China: simultaneous detection of novel mutations RDL A296G and VGSC A1007T
Source: Front Cell Infect Microbiol. 2026 Mar 27;16:1798952. doi: 10.3389/fcimb.2026.1798952 (PMC13066222; doi:10.3389/fcimb.2026.1798952)
Supplement: Supplementary file 1 [file Table1.docx]

**Table S1. Haplotype sequence information of each gene with GenBank accession numbers.**

| Haplotype Sequence | GenBank accession numbers |  |
| --- | --- | --- |
|  |  |  |
| VGSCH1 (1014L) | PX893157 |  |
| VGSCH2 (1014L) | PX893158 |  |
| VGSCH3 (1014L) | PX893159 |  |
| VGSCH4 (1014L) | PX893160 |  |
| VGSCH5 (1014L) | PX893161 |  |
| VGSCH6 (1014F) | PX893162 |  |
| VGSCH7 (1014F) | PX893163 |  |
| VGSCH8 (1014F+1007T) | PX893164 |  |
| VGSCH9 (1014S) | PX893165 |  |
| AChEH1 (119G) | PX893166 |  |
| AChEH2 (119S) | PX893167 |  |
| AChEH3 (119G) | PX893168 |  |
| AChEH4 (119G) | PX893169 |  |
| AChEH5 (119G) | PX893170 |  |
| AChEH6 (119G) | PX893171 |  |
| AChEH7 (119G) | PX893172 |  |
| AChEH8 (119G) | PX893173 |  |
| AChEH9 (119G) | PX893174 |  |
| AChEH10 (119G) | PX893175 |  |
| AChEH11 (119G) | PX893176 |  |
| AChEH12 (119G) | PX893177 |  |
| AChEH13 (119G) | PX893178 |  |
| AChEH14 (119G) | PX893179 |  |
| AChEH15 (119G) | PX893180 |  |
| AChEH16 (119G) | PX893181 |  |
| RdlH1 (296A) | PX893182 |  |
| RdlH2 (296A) | PX893183 |  |
| RdlH3 (296A) | PX893184 |  |
| RdlH4 (296G) | PX893185 |  |
| RdlH5 (296S) | PX893186 |  |
